# Recreational Marijuana Legalization and Workplace Injuries Among Younger Workers

**DOI:** 10.1001/jamahealthforum.2023.5438

**Published:** 2024-02-23

**Authors:** Ling Li, Yang Liang, Joseph J. Sabia, Dhaval M. Dave

**Affiliations:** 1 Department of Economics, University of Wisconsin-Parkside, Somers, Wisconsin; 2 Center for Health Economics & Policy Studies, San Diego State University, San Diego, California; 3 Department of Economics, Bentley University, Waltham, Massachusetts

## Abstract

This case-control study uses state-by-year workplace injury data to assess recreational marijuana legalization adoption and workplace injuries among younger workers aged 20 to 34 years.

## Introduction

Since 2012, 24 states and Washington, DC, have legalized the possession and sale of small quantities of marijuana for recreational purposes. Recreational marijuana laws (RMLs) are associated with higher adult marijuana use,^1^ but their broader impacts in terms of health and safety remain unexplored.^2^

This study assesses the association between RML adoption and workplace injuries in younger workers aged 20 to 34 years. A priori, this association may be directionally ambiguous: RMLs may be associated with greater workplace injuries if increased marijuana use diminishes workers’ cognitive functioning or acts as a gateway to harder drugs,^2,3^ or RMLs may be associated with fewer injuries if marijuana treats pain that contributes to workplace accidents or induces substitution away from alcohol or opioids.^2,4^

## Methods

We used state-by-year workplace injury data from the Bureau of Labor Statistics Survey of Occupational Injuries and Illnesses.^5^ From 2006 to 2020, 13 states adopted RMLs and 10 states permitted recreational sales (eTable in Supplement 1). Our analysis sample comprises 632 state-year observations with information on age-specific workplace injuries. Outcomes were the natural log of workplace injuries per 100 full-time equivalent workers and per 100 persons (both aged 20-34 years). Institutional review board approval was not required per the Common Rule (45 CFR §46.104) because of the use of secondary and deidentified data.

We applied a difference-in-differences multivariate regression framework, adjusting for dummy variables for each state and year, presence of a medical marijuana law, presence of a marijuana decriminalization or depenalization law, macroeconomic conditions, demographic characteristics, substance use policies, tobacco policies, per capita COVID-19 deaths, and the maximum workers compensation benefit (eMethods in Supplement 1). The estimated associations between RMLs and workplace injuries (reported as β coefficients) are translated to percentage changes by calculating *(e^β^* *–* *1)* *×* *100.* Estimates were interpreted as statistically significant if *P* value <.05, based on 2-sided hypothesis tests.

## Results

The Table reports associations between RMLs and workplace injuries. Controlling for marijuana policies, state dummies, and year dummies, RML adoption was associated with a statistically significant 12.9% increase (β, 0.121; 95% CI, 0.047-0.156; *P* = .002) in workplace injuries per 100 full-time workers. Adjusting for all covariates, the estimated increase is 9.6% (β, 0.092; 95% CI, 0.027-0.157; *P* = .01). With respect to the injury rate per 100 persons, RML adoption was associated with an 8.4% increase (β, 0.081; 95% CI, 0.009-0.152; *P* = .03) in the fully adjusted specification.

**Table.** Recreational Marijuana Laws (RMLs) and Natural Log of Workplace Injuries From 632 State-Year Observations

Dependent variable	Association between RML adoption and natural log of workplace injuries, β (95% CI)^a^					
	Model 1		Model 2			
	RML^b^	*P* value	RML with sales allowed^b^	*P* value	RMLs with no sales allowed^b^	*P* value
Natural log of workplace injuries per 100 full-time workers, aged 20-34 y						
Partially adjusted model^c^	0.121 (0.047 to 0.156)	.002	0.133 (0.045 to 0.220)	.004	0.090 (0.009 to 0.171)	.03
Fully adjusted model^d^	0.092 (0.027 to 0.157)	.01	0.112 (0.031 to 0.193)	.01	0.049 (−0.021 to 0.118)	.17
Natural log of workplace injuries per 100 full-time persons, aged 20-34 y						
Partially adjusted model^c^	0.130 (0.061 to 0.200)	<.001	0.140 (0.054 to 0.225)	.002	0.105 (0.050 to 0.161)	<.001
Fully adjusted model^d^	0.081 (0.009 to 0.152)	.03	0.095 (0.006 to 0.185)	.04	0.049 (−0.012 to 0.110)	.12

^a^ Weighted regressions are estimated using 2-way fixed-effects model where the dependent variables are the natural log of workplace injuries per 100 full-time equivalent workers aged 20 to 34 years (model 1), and the natural log of workplace injuries per 100 persons aged 20 to 34 years (model 2).

^b^ The 95% CIs are calculated with the SEs clustered at the state level.

^c^ The partially adjusted model includes dummies for each state, dummies for each year, medical marijuana laws, and marijuana decriminalization or depenalization laws.

^d^ The fully adjusted model extends the partially adjusted model by including state unemployment rate, and natural log of per capita personal income, share of state population that are Black, Hispanic, and female, the presence of medical marijuana laws, marijuana decriminalization laws, Good Samaritan drug laws, naloxone access laws, prescription drug monitoring programs, state minimum wage, state maximum monthly cash benefit for workers’ compensation recipients, cigarette tax, presence of e-cigarette tax, beer tax, Tobacco 21-law, and annual number of cumulative COVID-19 deaths per capita.

In Figure, A, the RML indicator was replaced with its leads and lags. Prior to RML adoption, no association with workplace injuries was found. Two and 3 years postadoption, injuries were significantly higher. Event-study estimates (Figure, B), which adjust for potential bias from staggered policy adoption, yielded similar results.

**Figure.**
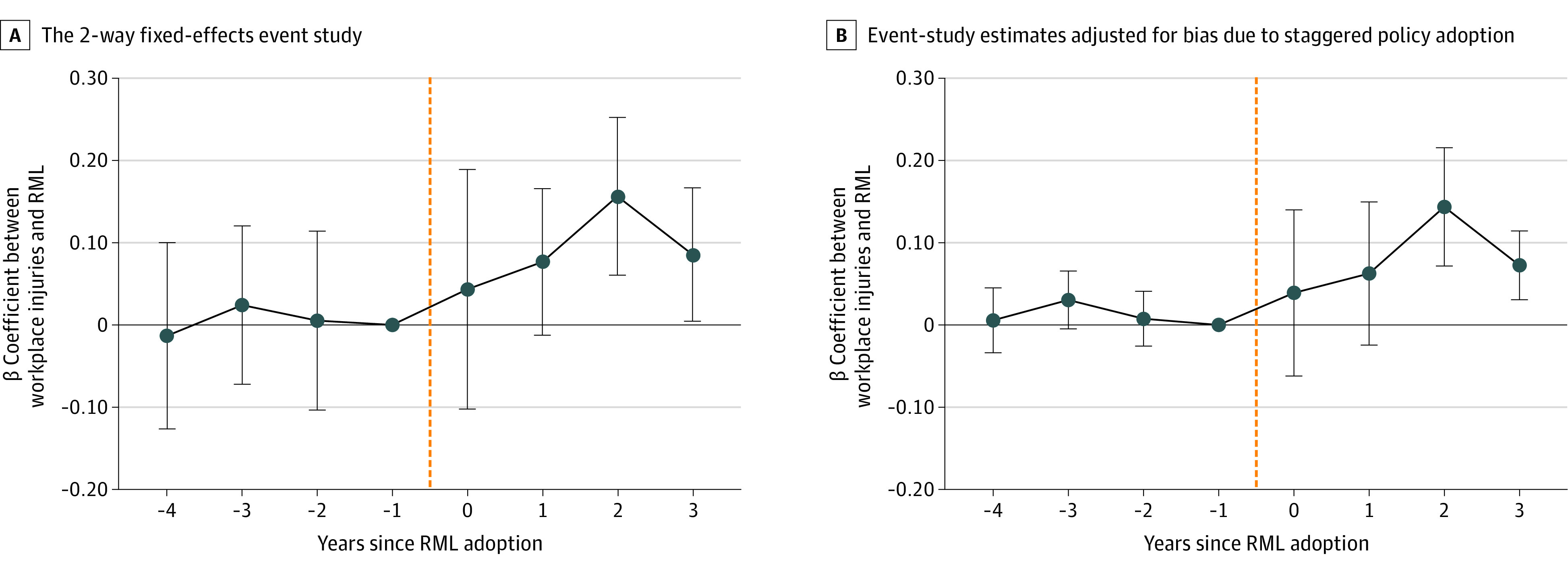
Event-Study Analysis of Recreational Marijuana Law (RML) Adoption and Workplace Injury Rates Vertical error bars indicate 95% CIs. The orange dashed line delineates the period before and after the enactment of RML.

The Table also differentiates RMLs that allow recreational sales (ie, dispensaries). In the fully adjusted regressions, recreational sales were associated with an 11.9% increase (β, 0.112; 95% CI, 0.031-0.193; *P* = .01) in workplace injuries per 100 full-time workers and a 10.0% increase (β, 0.095; 95% CI, 0.006-0.185; *P* = .04) in injuries per 100 persons; RMLs that did not allow sales were not associated with injuries.

## Discussion

In this study, RMLs that allow recreational marijuana sales were associated with a 10% increase in workplace injuries among individuals aged 20 to 34 years. Assuming that increased marijuana use^1,6^ was the primary channel through which RMLs affected workplace injuries implies an injury elasticity with respect to marijuana of 0.2 to 0.4. Our findings are consistent with the hypothesis that recreational marijuana impedes cognitive function and care among younger workers. This differs from older workers, for whom prior research uncovered a decline in workers’ compensation benefit receipt and nontraumatic injuries following RML adoption.^4^ Marijuana access may have differentially helped older workers manage pain. Thus, responses could be heterogeneous across differently aged workers. Because less than 4 years have elapsed on average post-RML adoption over our sample, our study is limited to assessing shorter- and medium-run outcomes.

## Notes

Supplement 1. **eMethods.** Data and Empirical Model **eTable.** Effective Dates of Recreational Marijuana Laws (RMLs) Adopted Between January 2012 and December 2020

Supplement 2.
Data Sharing Statement
