# Supplementary material for: Recreational Marijuana Legalization and Workplace Injuries Among Younger Workers
Source: JAMA Health Forum. 2024 Feb 23;5(2):e235438. doi: 10.1001/jamahealthforum.2023.5438 (PMC10891478; doi:10.1001/jamahealthforum.2023.5438)
Supplement: Supplement 1. — eMethods. Data and Empirical Model eTable. Effective Dates of Recreational Marijuana Laws (RMLs) Adopted Between January 2012 and December 2020 [file jamahealthforum-e235438-s001.pdf]

Supplemental Online Content

Li L, Liang Y, Sabia JJ, Dave DM. Recreational marijuana legalization and workplace injuries among younger workers. *JAMA Health Forum.* 2024;5(2):e235438. doi:10.1001/jamahealthforum.2023.5438

eMethods. Data and Empirical Model

eTable. Effective Dates of Recreational Marijuana Laws (RMLs) Adopted Between January 2012 and December 2020

This supplemental material has been provided by the authors to give readers additional information about their work.

© 2024 Li et al. *JAMA Health Forum.*

Online Supplement

Data

The data on workplace injury rate is from the Survey of Occupational Injuries and Illnesses (SOII) conducted by the Bureau of Labor Statistics (BLS). SOII is an annual survey that mandates reports of occupational injuries and illnesses by a sample of approximately 200,000 employers. The sample includes establishments in the private industry and the state and local government. Self-employed, farms with fewer than 11 employees, private households, and federal government agencies are excluded. Our analysis focuses on injury rates in the private sector. The data can be accessed from https://www.bls.gov/iif/data.htm. The data at the state level are available among 44 states and the District of Columbia. Our analysis sample includes 632 state-by-year observations from 2006 to 2020.[1] The data on effective dates of RMLs and the dates recreational marijuana sales were allowed are collected from Anderson and Rees (2023) and listed below in eTable.

eTable. Effective Dates of Recreational Marijuana Laws (RMLs) Adopted Between January 2012 and December 2020

	RML Effective Date	Date Recreational Sales Allowed
Alaska	2/24/2015	10/29/2016
Arizona	11/30/2020	
California	11/9/2016	1/1/2018
Colorado	12/10/2012	1/1/2014
D.C.	2/26/2015	2/26/2015
Illinois	1/1/2020	1/1/2020
Maine	1/31/2017	
Massachusetts	12/15/2016	11/20/2018
Michigan	12/6/2018	12/1/2019
Nevada	1/1/2017	7/1/2017
Oregon	7/1/2015	10/1/2015
Vermont	7/1/2018	
Washington	12/6/2012	7/8/2014

Source: Anderson and Rees (2023)

[1] State-years where age-specific workplace injuries are not reported to the BLS include Colorado, Florida (2011-2020), Georgia (2020), Idaho, Massachusetts (2009), Mississippi, New Hampshire, North Dakota, Ohio (2006-2011), Oklahoma (2013-2019), Pennsylvania (2006-2010), Rhode Island (2008-2020), and South Dakota.

© 2024 Li et al. *JAMA Health Forum.*

Empirical Model

Our main empirical approach is a two-way fixed effects (TWFE) "difference-in-differences" model. We estimate the following regression equation:

$$Y_{st} = \alpha + \beta RML_{st} + \boldsymbol{Z'}_{st}\boldsymbol{\alpha} + \theta_s + \tau_t + \epsilon_{st} \qquad \text{(a)}$$

Y_{st} is the natural log of the workplace injury rate per 100 full-time workers ages 20-34 (or per 100 persons ages 20-34) in state s and year t, RML_{st} is the share of the year *t* (ranging from 0 to 1) that a recreational marijuana law is in effect in state *s*, and $\boldsymbol{Z}_{st}$ is a vector of control variables, including

- marijuana policy controls (presence of medical marijuana laws, marijuana decriminalization laws)
- macroeconomic controls (state unemployment rate and per capita personal income),
- demographic controls (share of state population that are black, Hispanic, and female),
- other drug policy controls (Good Samaritan drug laws, Naloxone access laws, and prescription drug monitoring programs),
- labor and workplace safety policy controls (state minimum wage and maximum monthly cash benefit for workers' compensation recipients),
- cigarette and alcohol policy (cigarette tax, presence of e-cigarette tax, beer tax, Tobacco-21 law), and
- COVID-19 controls (number of cumulative covid deaths per capita).

Finally, θ_s is a set of time-invariant state dummies (for the 45 states available in our sample) and τ_t is a set of state-invariant year dummies (for the 15 years in our sample). Regressions are weighted by the state-specific number of workers (or population) ages 20-34. Standard errors are clustered at the state level (Bertrand et al. 2004).

The event study figures presented the dynamic effect of RMLs on workplace injury rate. The figures are generated by estimating the following equation:

$$Y_{st} = \delta + \textstyle\sum_{j \neq -1} \pi_j D_{st}^{j} + \boldsymbol{Z'}_{st}\boldsymbol{\gamma} + \eta_s + \rho_t + \mu_{st} \qquad \text{(b)}$$

where D_{st}^{τ} are treatment indicators of leads and lags of the RMLs, which equals 1 for an event (RML enactment) happening *j* periods away from *t*, and treatment indicators are binned at the "endpoints" (the last open-ended lead and lag variables). The vector π_j denotes the coefficients on the treatment effect, with the reference period being *j-1*, the year prior to RML enactment.

We utilize the methods of Gardner (2021) to address the potential bias in TWFE models due to heterogeneous and dynamic treatment effects in staggered policy adoption. The first stage of the procedure consists of a regression of outcomes on state and year fixed effects, estimated using the subsample of untreated observations. In the second stage, the estimated state and year effects are subtracted from observed outcomes, and these adjusted outcomes are regressed on treatment.

© 2024 Li et al. *JAMA Health Forum.*

eReferences

Bertrand, M., Duflo, E. and Mullainathan, S., 2004. How much should we trust differences-in-differences estimates?. *Quarterly Journal of Economics*, *119*(1), pp.249-275.

Gardner, J., 2022. Two-stage differences in differences. arXiv preprint arXiv:2207.05943.

© 2024 Li et al. *JAMA Health Forum.*
