# Supplementary material for: Recreational Marijuana Legalization and Workplace Injuries Among Younger Workers
Source: JAMA Health Forum. 2024 Feb 23;5(2):e235438. doi: 10.1001/jamahealthforum.2023.5438 (PMC10891478; doi:10.1001/jamahealthforum.2023.5438)
Supplement: Supplement 2. — Data Sharing Statement [file jamahealthforum-e235438-s002.pdf]

Data Sharing Statement

Li. Recreational Marijuana Legalization and Workplace Injuries Among Younger Workers. *JAMA Health Forum*. Published February 23, 2024. doi:10.1001/jamahealthforum.2023.5438

Data
Data available: Yes
Data types: Deidentified participant data
How to access data: Publicly available from Bureau of Labor Statistics
When available: With publication

Supporting Documents
Document types: Statistical/analytic code
How to access documents: Email to author Yang Liang, yang.liang@sdsu.edu
When available: With publication

Additional Information
Who can access the data: Anyone wishing to replicate.
Types of analyses: Regression analysis.
Mechanisms of data availability: After emailing request.
